# Erosion of the Teeth Considered as a Retrospection Sign of Infantile Eclampsia

**Published:** 1883-03

**Authors:** 


### Erosion of the Teeth Considered as a Retrospection Sign of Infantile Eclampsia.

A paper on this subject was read before the International Medical Congress, held in London, August, 1881, by Dr. Magitot, of Paris.

Upon that paper was had the following discussion, which presents the views of several gentlemen of the profession upon the principal points of the paper. It is a subject of much interest.

Mr. Moon, London: In discussing this subject, it may be taken for granted that all medical men will admit that hereditary syphilis is very apt to be productive of a deformity of certain among the permanent teeth; but there are not a few in the ranks of medicine and surgery who consider that such malformation may be due to other causes besides syphilis. Now this doubt is the outcome, I believe, of a widespread want of distinctness that prevails as to what a typical syphilitic tooth really is. Mr. Jonathan Hutchinson having, years ago, shown the way, it of course is comparatively easy for others to follow out details of this subject. The diagnostic value of a syphilitic tooth depends greatly on this fact, viz.: whether such malformation is distinctive and due to syphilis alone, or whether other causes may bring about the like malformation? Some years ago I collected cases and took models of a large number of cases bearing on this subject, and became thereby convinced that there is a special kind of malformation which may be depended on as indicating hereditary syphilis and nothing else. In order to properly appreciate divergences from the normal form of teeth, it is necessary that some features of what may be called the architecture of a normal tooth should be known. To elucidate this subject and to lessen the probability of my remarks wearying you, I have brought down some models and put up some drawings which will, I hope, clearly bring out the normal form of syphilitic teeth, and the shape of such teeth as are apt to be confused with the latter.

A description was then given of the differences between normal and syphilitic teeth, and the modes of distinguishing the latter from other malformed teeth. The different kinds of defective enamel formation were then touched on, and the question suggested that perhaps "mercurio-syphilitic tooth" might be the right term for certain teeth which presented a want of enamel over a semi-lunar space in the center of the first formed portion, leading often to the breaking down of the unprotected dentine and the formation of a semi-lunar notch.

Mr. C. S. Tomes, London: exhibited a model of teeth presenting in a marked degree M. Magitot's "erosion," remarking that the history of the patient was perfectly known to him, and that no attack of convulsions had ever occurred. The patient had, however, suffered from a severe attack of inflammatory croup at such an age as to exactly correspond with the lesion of the teeth. During this attack she was salivated.

Mr. Coleman, Streatham: It was my good fortune to be a colleague of Mr. J. Hutchinson at the period when that gentleman's attention was first directed to this subject. I thus had the opportunity of seeing a considerable number of Mr. Hutchinson's earliest cases, and to his first communication—viz., that to the Pathological Society—was appended a report by me of the peculiarities such teeth presented. I confess I was in the first instance a skeptic with regard to the syphilitic origin of these teeth, but as time wore on I can only come to the conclusion of the correctness of Mr. Hutchinson's views. My observations have now been extended over some twenty years, and from holding hospital appointments I have had large opportunities of inspecting the teeth of all classes, and my firm and settled conviction is, that between cause and effect there can not in my mind be the least doubt. Persons the subjects of inherited syphilis may have well-formed and excellent teeth, but in my belief no person can have the type of teeth described by Mr. Hutchinson who has not inherited syphilis.

Dr. Blache, Paris: In a paper which I published in 1879 on "Malformation of Teeth in Children as a Symptom of Hereditary Syphilis," I quoted five instances of cases having reference to the first dentition. In taking part in the interesting discussion opened to-day, I claim to contribute towards the establishment of the fact of "erosion" taking place in the milk-teeth, not only in cases of syphilis or in the children of syphilitic parents, but sometimes also quite independently of this condition. Thus I have had under observation for four years a child at whose birth I was present, and whose father and mother I know and believe to be absolutely free from syphilitic disease. This child has marks of "erosion" on its lower incisor teeth and on the two canines, which I can not refer either to syphilis or to those convulsions, which my friend and colleague M. Magitot deems to be the origin of this affection.

Dr. Hayward, Liverpool: I wish to contribute a practical case. The parents were perfectly healthy, having six quite healthy children; after this the father had occasional intercourse with a lady with tertiary syphilis during daytime, and at night with his own wife. After this twins were born; both with very definite signs of inherited syphilis, one dying within three months, the other struggling through, but absolutely without mercurial treatment. Its primary teeth were black and soft, the permanent incisors some defective of dentine, with small cutting edges and large at the neck, and others defective of enamel and showing honeycomb appearance. There was scarcely any medicinal treatment, and certainly not mercurial. The child never had convulsions or any similar disease; only a great liability to bronchitis. This would show marked teeth originating without nervous disease or mercury, but certainly from syphilis; this view being further supported by the fact that both parents afterwards had cutaneous syphilis.

Dr. Dally, Paris: It is many years since M. Magitot first acquainted me with his views on the origin of erosions of the teeth. Since then I have made extended inquiries in my outpatient practice amongst the children whom I attend for deformities of the organs of locomotion, and my inquiries have very rarely failed to elicit a history of convulsions. At the same time I would add that I have never been able to entirely exclude the suspicion of syphilis. The difficulty is that people are ready enough to admit convulsions, but will always endeavor to conceal syphilis. Why must I exclude one of these two causes? Because hereditary syphilis does not cause disturbances of the nerve centers? Convulsions are only a symptom. There is some morbid cause behind them, and do we know of any *causa morborum* more active, more profound, and more protean than syphilis? Erosion of the teeth is evidence of a violent and passing disturbance of general nutrition. True; and the general conclusion which, as it appears to me, is to be deduced from this discussion is, that all the causes which are capable of interfering with general nutrition—accidental and physiological causes included—are capable of producing erosions of the teeth.

Mr. Hutchinson, London: I avail myself with some pleasure of the opportunity which this debate affords me of attempting to correct some misapprehensions in reference to the clinical symptology of the teeth. M. Magitot makes the denial that honeycomb teeth or eroded teeth are indicitive of inherited syphilis one of the main points of his paper, and he seems to think that in saying this he is controverting an opinion of mine. But in point of fact we are in accord, for this is what all along I have most carefully asserted. The honeycombed eroded teeth have always for me been indicitive of infantile stomatitis (mercurial or other), and never of syphilis. I am aware that mistakes have been made, and that others besides M. Magitot have supposed that this condition of erosion was what I had described as indicating syphilis. Thus, for instance, most of the casts which M. Parrot shows in our museum as syphilitic are not what I should regard as syphilitic at all, but simply "mercurial." This error has been the more frequent because, as a fact, we very frequently—indeed I may say usually—meet with the malformations due to syphilis and those due to stomatitis in the same mouth; for most syphilitic children have taken mercury in infancy. But I can not myself plead guilty to any responsibility for such mistakes, for both in pictorial representation and description, I have always carefully noted the differences and distinguished the two conditions. Even where the two are co-existent there is no real difficulty in discrimination. Permit me to repeat what I have said over and over again on former occasions, that the dental malformations which denote syphilis are not of the nature of erosion, but consist in very peculiar arrests of development. The only teeth to which I venture to attach much importance are the upper central incisors of the permanent set. In these an arrest in the growth of the middle denticle, leaving a single central notch, is the commonest and most trustworthy condition, but, in addition to it, there is usually a dwarfing of the tooth in all its dimensions, and sometimes a screw-driver form of tooth is almost as characteristic as a notched one. The defects are usually symmetrical, but sometimes not so. Peculiarities are often to be observed in the other teeth, and are often of much help in corroboration of diagnosis, but in the abscence of the peculiarity described in the upper central incisors they are not to be trusted, and for fear of occasioning more mistakes I prefer not to allude to them further. Whilst these malformations of the upper central incisors (notches and dwarfing) are the characteristics of syphilitic teeth, those which imply former stomatitis consist chiefly in defective formation of the enamel. The teeth which show these most constantly are the first permanent molars, the two bicuspids being remarkable for their exemption. The explanation of this is easily given if we remember the dates of the calcification of the several teeth. The first permanent molars may be regarded as the test teeth as regards infantile stomatitis, just as the central incisors are for syphilis, but they are by no means the only teeth affected. Next to them in importance come the incisors, which are almost as constantly pitted, eroded, and of bad color, often showing a transverse furrow which crosses all the teeth at the same level. M. Magitot, if I have understood him correctly, inclines to the beliaf that these enamel defects are the consequences not so much of stomatitis as of some interference with development brought about through the nervous system in connection either with infantile convulsions or severe illness in early life. He explains them as we explain the furrows on the nails, so often seen as the record of past disturbances of health. It may be that to some extent he is right. I think it very probable that he is. I have myself long ago published facts bearing upon this very point, and have discussed in some detail the question as to whether, in the cases of enamel defects, so frequently seen with lamellar cataract, and in those who have had convulsions, the latter ought to be regarded as the direct cause of the former. The conclusion to which I have arrived is, that even in cases in which defects are in association with neurosis, still there almost invariably intervenes a stage of inflammation which is the immediate cause of the defects. So it is very possible that some stomatitis teeth may be caused by mercurial stomatitis or that of thrush, and that others may have been induced by a stomatitis due to nervous influences. Still, in both the local process which causes the defect is inflammation. Unquestionably, in the case of the nails, local diseases, certainly inflammatory eczema, etc., produce markings just like those which follow general illness. We are now, Mr. President, I think, in a fair position to discuss the questions, Are the teeth which have been described as syphilitic really due to syphilis? are those which are assigned to stomatitis really in association with that cause? The evidence on the latter point is of a far less conclusive character than that as to the former. I have seen many sets of so-called mercurial or stomatitis teeth in which I could get no evidence that mercury had been given, or that any form of infantile illness had been experienced likely to be attended by stomatitis. Still, although not uncommon, this class of facts has been, in my experience, exceptional, and those which have illustrated what I believe to be the rule have been far more abundant. About the time that we were first noticing syphilitic teeth, and were busy examining the mouths of our young patients, the observation was made that in most cases of infantile cataract the teeth were very defective. I do not know whether Professor Horner, of Zurich, Mr. Teale, of Leeds, or myself first noted this point, but we were all at work on it together. To Professor Horner, I believe, belongs the credit of having drawn attention to the fact that the kind of infantile cataract usually so attended was almost always "lamellar," and that there was usually also a history of convulsions in infancy. Then comes the problem as to the mutual relationship between these three conditions--convulsions in infancy, lamellar cataract, and defectively developed teeth. Upon this I wrote a paper which was published in the Pathological Society's *Transactions* in 1875.* I must not weary you by recapitulating the arguments of that paper, more especially as it is accessible to all who feel sufficient interest in the subject to refer to it. Briefly, my conclusion was, that the cataract was due to the convulsions, and the dental defect to the mercury given for the convulsions. It may easily be seen that I was to some extent wrong on this latter point, and I am quite willing to join with M. Magitot in the suspicion that the illness coincident with the convulsions may, in some cases without the help of mercury, produce defects in the enamel, etc. It is clear that we have here an interesting field for further observation. Let those who have the opportunities for knowing the facts accurately, that is, without trusting to heresay evidence, tell us whether convulsions without mercurial treatment and without any form of stomatitis are often followed by defective teeth. Conversely let those who possess facts as to children who, during the first year, have taken much mercury (as "teething powders"

* Transactions of the Pathological Society of London, Vol. XXVI. p. 235.

or otherwise), tell us whether in such, defective development of the enamel is ever absent in the permanent teeth. There must be plenty of facts of both classes extant if we could only get at them. That mercury given in infancy is one of the causes of eroded teeth I have not the least doubt. Many very convincing facts have occurred within my own observations. My friend, Mr. Moon, and others have also carefully investigated cases of this kind, and have arrived at the same conclusion. Here, then, I leave this part of our subject; it requires further work, but the main conclusions which I have formerly published are, I still believe, well sustained. Respecting the so-called "syphilitic" teeth, I am not prepared to admit the slightest doubt or hesitation. It is now more than twenty years since I submitted to the Pathological Society, and through it to the profession, the evidence which I had obtained on this point. Since then I have made daily use of these conditions as an aid in diagnosis, and their value has been acknowledged by a host of independent observers in all parts of the world. Indeed I may, I think, without presumption, claim that considerable advances in clinical knowledge have been made in various departments by their aid. Almost simultaneously with their recognition came the observation that "interstitial keratitis," when well marked and symmetrical, may be regarded as certainly syphilitic. It had formerly been always called "scrofulous." Choroiditis desseminata, a peculiar form of deafness, and a special form of rapidly destructive "lupus," were also soon afterwards assigned with confidence to their proper category. More recently, partly by the evidence of such affections as those just mentioned, but more particularly by that given by the teeth, various observers—Dr. Hughlings Jackson, Dr. Barlow, Dr. Allen Sturge, Dr. Down, and others—have recognized rare forms of disease of the nervous system as certainly due to inherited taint. A large group of bone diseases have in like manner, and on like evidence, been transferred from struma and rickets to syphilis. I produced yesterday, at the London Hospital, for M. Magitot's inspection, about a dozen examples of well-marked syphilitic teeth, occurring in association with one or other, or with several of the maladies I have enumerated.

Neither these maladies nor the teeth which have helped us to their recognition are, in certain departments of practice at all uncommon; and I repeat, our clinical knowledge of the one rests to a large extent upon the trustworthiness of the other. Such being the case, and these facts having been in the main recognized, not only in England, but over the Continent, and in America, I submit that Dr. Magitot is a little late in the day when he comes to tell us that these malformations are not indications of inherited syphilis but of convulsions. I hope I shall not be considered over-sensitive if I admit to a feeling that it is a little hard to be called on now, after my conclusions have been so long admitted, to again go over the old ground of proof. I should have no right to resent this if it came from one who had studied my views, and was prepared with facts to refute them. Before I sit down, sir, let me fully admit that it is not always that the teeth of the subjects of inherited syphilis reveal the diathesis. In very many cases they are not malformed at all. In many others the malformations are too slight or too much mixed to be worthy of confidence, though still possibly very valuable by way of corroboration. The frequent coincidence of syphilitic with mercurial peculiarities has made it much more difficult for learners to obtain clear perceptions respecting them than would otherwise have been the case. The neglect to discriminate between the two has led to innumerable errors. Into this error both Professor Parrot and the author of this paper have, I hold, fallen, and hence the doubts expressed by the latter.

Dr. Quinet, Brussels: There is no doubt that M. Parrot and Mr. Hutchinson hold different views as regards the influence of hereditary syphilis on the temporary teeth. It is proved by the facts of surgery that this diathesis, which so often kills the child in its mother's womb, and leaves its traces on the thymus gland, on the lungs, and on the liver, must also necessarily leave its mark upon the temporary teeth. Animals also sometimes present in their teeth the marks of blind erosion, as in the oturia gulata and the oturia stellaris in the Museum of the Royal College of Surgeons. Lastly, I recognize a general diathesis—rachitis scrofula —which has the same influence on the dental organs, an influence slow and prolonged in its action, and leaving its traces in the entire organs, and especially characterized by uniformity.

Professor Parrot, Paris: I shall answer the argument of M. Magitot, who rejects the syphilitic origin of dental atrophy, and adopts the "convulsive" theory, by three remarks. Firstly, I would call attention to the *systematic* nature of the dental change, certain teeth being affected, and others never. Thus all the teeth of the first dentition may be affected, and among those of the second dentition, the incisors, the canines, and the first molars; while the second and third molars never suffer, and the premolars but rarely, as I have never seen an example. Why should certain teeth have such an immunity? Convulsions take place at all times of infantile life, so all the teeth ought to be affected. Do convulsions or eclampsia take place in intra-uterine life? We do not know anything on this matter, but it is not very probable. But it is during intra-uterine life that the development of the caps of dentine of the first dentition takes place, and indeed also of several of those of the second dentition. It would therefore follow that during this time there must be numerous convulsions. And, lastly, the extent of the affected dentine and enamel can not be regarded as agreeing with the relatively short duration of the convulsive attacks.

M. Magitot.—The first speaker upon my communication, Mr. Moon, attributes the different kinds of erosion to the action of mercury. It is a view I do not comprehend. If I understand the operation of mercury it is upon the gums, and not upon the teeth, that it acts, and inflammation of the gums is capable in certain cases of producing marked impressions upon the teeth. This is quite a different lesion, and has nothing in common with that to which the paper refers. It seems to me that there is some confusion in the opinions presented by Mr. Moon in reference to the real erosion which is congenital, and certain pathological alterations of the crown which are shown on the border of the inflamed gum. My friend, Mr. C. S. Tomes, defends his former opinion in which he attributes the erosion to the various affections of childhood. I have long enough urged this explanation and so will not again refer to it: the essential condition necessary for the production of erosion is *the rapid invasion*, followed by the same rapid cessation which characterizes the affection, perhaps the cause of erosion ; it is this peculiarity which brings or causes the prompt interruption between the healthy region of the crown and the defective part. It is the nature of the intercurrent affection which must be at the same time sufficiently severe to bring a perturbation or suppression in the formation of the tissues, and sufficiently general to produce this difficulty of nutrition. These conditions, if we will trace them to their origin, will be found more or less directly connected with eclampsia. The precise nature of which, though not yet understood, certainly proceeds from some peculiar condition of the nervous system ; a condition always grave in character, and whose convulsive crises so sudden and impulsive are the outward manifestation.

This criticism also applies with equal force to Mr. Dally whose opinion is nearly allied to that of Mr. Tomes. As to Mr. Coleman, whose ideas are the same as those of Mr. Hutchinson, my reply will be the same as that to the author of the doctrine, Mr. Hutchinson himself. Now, the learned physician of the Hospital of London does not recognize the characteristic alterations of the lesions in the specimens I present, which, according to his views, are of hereditary syphilis, these which accompany interstitial karatiesis, an affection essentially syphalitic. The only form of of syphalitic affection, according to Mr. Hutchinson, is that exhibited by the deeply notched teeth. It, nevertheless, seems not possible to refuse to admit that all varieties of erosion are of the same nature, and that, consequently, if the hereditary syphilis can produce certain forms it could just as well produce others. The only difference being the time of the invasion and the territory of the teeth affected. Now, I produce here a considerable number of the various forms of erosions, those of Mr. Hutchinson as well as the others, and in their close relation to convulsions. If Mr. Hutchinson persists to regard as syphilitic the notched teeth, I will ask him to consider two facts. The first to prove that these patients have not in their childhood been affected by eclampsia ; the second to show that the same patients are really hereditary syphilitic subjects. Now, Mr. Hutchinson has not and cannot have a definite knowledge of the former condition of many of his patients in respect to eclampsia. Those who consult him in the hospitals are persons of whom it is impossible to obtain accurate and definite information in respect to infant and childhood life. I have certainly observed in many cases, that Mr. Hutchinson has kindly showed me, the co existence of dental alteration and interstitial keratitis, but from this point to the general etiology of these cases there seems to to be a wide distance.

There is still another question to be decided, viz: that of the syphilitic nature of the parenchymatous keratitis itself which certain writers consider as a scrofulous manifestation. Now, the identity of the two diatheses is perhaps not sufficiently demonstrated.

These diverse observations are naturally addressed to Mr. Parrat, to whom I owe, nevertheless, an answer upon many particular points. Mr. Parrot asks me how I explain that the temporary teeth are so seldom subject to erosion, and that certain permanent teeth (second bicuspid and second and third molars) are never.

The first explanation is very simple. The temporary teeth do not accomplish their complete evolution before birth and the diagram which I have shown indicates for each tooth the height of its cap of dentine at this date. Now, that the morbid intervention supervenes in the first ranks of life the erosion will occupy upon the crowns of the temporary teeth, a point somewhat distant from the cutting edge, and it is also true that the milk teeth never present erosions in the form of a scratch, but always in deep furrows.

It is true nevertheless that certain temporary teeth manifestly present traces of analogous alterations, if not identical to erosion, and it is on account of these cases that Mr. Blache asks if I believe in a morbid, inter-uterine influence. Apparently all lesion of the temporary teeth implies a cause altogether maternal. But, what is this cause? Shall we say, it is eclampsia? I would not dare to affirm it here, though I thought at one time to have discovered a case of this kind in a mother who died during parturition, having been pregnant eight months. This child had signs of erosion upon the temporary cuspids. But what is easy to comprehend is that at this fatal period the disturbing influences of the interfollicular evolution of a tooth are yet now more easy than later, and that the maternal influence upon the child may be quite admissible.

Besides, I have said in the paper already read, that many cases of erosion of temporary teeth, presented by Mr. Parrot, had seemed to me to be post mortem alterations. Now, if certain permanent teeth are never attacked by erosions, it is, as I have remarked, that the state of their development is not subject to it.

In a case of erosion in the form of furrows, of two first premolars, we have found eclampsia, at three and a half years from the time of the development of the crown. The second molars finally make their appearance later. According to the dates we have fixed they are not liable to be affected by eclampsia, the appearance of which does not occur later than the fourth or fifth year. Mr. Parrot insists, with reason, upon the necessity in this research for the causes of dental erosion, for systematizing the facts which are found in connection with it. It is precisely in this direction that I have directed all my efforts; only while Mr. Parrot systematizes with the view of syphilis, I systematize with the view of eclampsia, and it is for this cause that I cannot understand how hereditary syphilis, by its slow and continued action, can produce, with this distinctness of furrow and pit, the alteration that I have described.

